# Effects of exercise on myokine gene expression in horse skeletal muscles

**DOI:** 10.5713/ajas.18.0375

**Published:** 2018-09-13

**Authors:** Hyo Gun Lee, Jae-Young Choi, Jung-Woong Park, Tae Sub Park, Ki-Duk Song, Donghyun Shin, Byung-Wook Cho

**Affiliations:** 1 Department of Animal Science, College of Natural Resources and Life Sciences, Pusan National University, Miryang 50463, Korea; 2 Graduate School of International Agricultural Technology and Institute of Green-Bio Science and Technology, Seoul National University, Pyeongchang 25354, Korea; 3 Department of Animal Biotechnology, Chonbuk National, University, Jeonju 54896, Korea

**Keywords:** Exercise, Horse Skeletal Tissue, Horse Skeletal Muscle Cells, Myokine

## Abstract

**Objective:**

To examine the regulatory effects of exercise on myokine expression in horse skeletal muscle cells, we compared the expression of several myokine genes (interleukin 6 [*IL-6*], *IL-8*, chemokine [*C-X-C motif*] ligand 2 [*CXCL2*], and chemokine [*C-C motif*] ligand 4 [*CCL4*]) after a single bout of exercise in horses. Furthermore, to establish *in vitro* systems for the validation of exercise effects, we cultured horse skeletal muscle cells and confirmed the expression of these genes after treatment with hydrogen peroxide.

**Methods:**

The mRNA expression of *IL-6*, *IL-8*, *CXCL2*, and *CCL4* after exercise in skeletal muscle tissue was confirmed using quantitative-reverse transcriptase polymerase chain reactions (qRT-PCR). We then extracted horse muscle cells from the skeletal muscle tissue of a neonatal Thoroughbred. Myokine expression after hydrogen peroxide treatments was confirmed using qRT-PCR in horse skeletal muscle cells.

**Results:**

*IL-6*, *IL-8*, *CXCL2*, and *CCL4* expression in Thoroughbred and Jeju horse skeletal muscles significantly increased after exercise. We stably maintained horse skeletal muscle cells in culture and confirmed the expression of the myogenic marker, myoblast determination protein (*MyoD*). Moreover, myokine expression was validated using hydrogen peroxide (H_2_O_2_)-treated horse skeletal muscle cells. The patterns of myokine expression in muscle cells were found to be similar to those observed in skeletal muscle tissue.

**Conclusion:**

We confirmed that several myokines involved in inflammation were induced by exercise in horse skeletal muscle tissue. In addition, we successfully cultured horse skeletal muscle cells and established an *in vitro* system to validate associated gene expression and function. This study will provide a valuable system for studying the function of exercise-related genes in the future.

## INTRODUCTION

The horse is a valuable model animal for studying the effects of exercise, because it is the most adaptive animal for exercise among livestock. Thoroughbreds are one of the most famous horse breeds in the horse racing industry, and they were specially bred for speed, endurance, and strength from the early 1700s. Through the selection of these characteristics, Thoroughbred has become a horse for racing [1]. The exercise characteristics of Thoroughbred are used as a biological model in the field of exercise physiology, and helped identify the molecular mechanisms of the adaptive responses to exercise [2].

To date, a number of genes related to exercise have been screened through high throughput analyses such as RNA sequencing [3] or microarrays [4]. A number of differentially expressed genes from genomic studies have been studied for functions in the stress response [2,5] and validated in horse muscles [6–8] Exercise-induced stress is considered one of the major stimuli for the adaptation to and improvement of physical performance of racing horses. Exercise induces endoplasmic reticulum, oxidative, and inflammatory stresses in muscle. After muscles contract during exercise, skeletal muscles produce a group of cytokines called myokines (interleukin 6 [*IL-6*], interleukin 6 [*IL-8*], chemokine [*C-C motif*] ligand 4 [*CCL4*], and chemokine [*C-X-C motif*] ligand 2 [*CXCL2*]), which function in metabolism, insulin action, and inflammatory responses [9–13]. Among these, *IL-6* is the first myokine to be secreted into circulation [14], and it acts as both a pro-inflammatory and an anti-inflammatory myokine. Plasma *IL-6* was locally expressed in skeletal muscle without muscle damage, and its concentration dramatically increased following exercise [10]. To date, *IL-6* is known to induce a variety of effects, including glucose and fat metabolism [15,16] and anti-inflammatory functions during exercise [17]. *IL-8* is a chemokine, which mainly functions in the chemotaxis of neutrophils. It is also expressed in skeletal muscle after exercise [11], and it has been hypothesized to play a role in angiogenesis within skeletal muscles [18]. After endurance racing in horses, *CXCL2*, also called macrophage inflammatory protein 2-α (*MIP2-α*), is upregulated in peripheral blood mononuclear cells [4]. In humans, *CXCL2* is significantly induced in exercising legs [19]. *CCL4* is a chemoattractant for a variety of immune-related cells [20]. It is highly expressed in skeletal muscles in response to pathological situations [21], and the concentration of *CCL4* increases following exercise [12]. It induces myoblast proliferation via G protein-coupled receptors, extracellular signal-regulated kinases 1/2, and mitogen-activated protein kinase pathway, and it may be involved in wound healing after muscle injury [22,23].

In this study, we examined the gene expression of myokines, including *IL-6*, *IL-8*, *CXCL2*, and *CCL4* in Thoroughbred and Jeju horse skeletal muscles before and after a single bout of exercise. Furthermore, we tested the myokine expression in primary muscle cells that were derived from Thoroughbred skeletal muscles in response to hydrogen peroxide (H_2_O_2_) treatment, which mimics oxidative stress *in vitro*. The results of this study could be valuable for the establishment of strategies that manage exercise-induced muscle damage in the equine industry.

## MATERIALS AND METHODS

### Study animals

Six horses were used in this study, and they were divided into two groups: Thoroughbred and Jeju horses. The Pusan National University-Institutional Animal Care and Use Committee approved the study design (Approval Number: PNU-2015-0864).

### Tissue sampling

Two stallions, one Thoroughbred mare, and three Jeju mares (aged 5 to 10 and weighing 500 to 700 kg) were used to obtain skeletal muscle samples before and after exercise. Exercise involved trotting at 13 km/h for 30 min and cantering through lunging and long-reining (circular bridge lunging). Skeletal muscle samples were collected from the triceps brachii of the right leg.

### Primary horse muscle cell culture

A skeletal muscle tissue biopsy was performed on the leg of a neonatal Thoroughbred. Horse skeletal muscle cells were maintained and sub-passaged in Medium 199 (Gibco, Grand Island, NY, USA) supplemented with 10% fetal bovine serum (FBS; Invitrogen, Carlsbad, CA, USA), 2% donor equine serum (DES; Hyclone, Carlsbad, CA, USA), and 1% antibiotic-antimycotic (ABAM; Invitrogen, USA). Medium 199 supplemented with 0.5% FBS and 1% ABAM was used as the differentiation medium. Horse skeletal muscle cells were cultured in a humidified atmosphere with 5% CO_2_ at 37°C. At approximately 70% to 80% confluence, cells were treated with 1 mM H_2_O_2_ (Junsei, Tokyo, Japan) and cultured for 6 h. Cells were gently washed twice with phosphate-buffered saline, and were then harvested using 0.05% trypsin-ethylenediaminetetraacetic acid (Welgene, Gyeongsan, Korea) to extract total RNA.

### RNA extraction and cDNA synthesis

Horse skeletal muscle tissue (~50 to 100 g) was crushed using a mortar, and ground muscle tissue was then dissolved using 1 mL TRIzol (Invitrogen, Karlsruhe, Germany). Next, 200 μL of chloroform was added to remove cells from the organic solvent, the mixture was shaken for 10 s, maintained at 4°C for 5 min, and centrifuged at 4°C for 15 min. The supernatant was removed and added to a new test tube, mixed with an equal amount of isopropanol, and maintained at 4°C for 15 min to collect RNA pellets. Isopropanol was removed from the solution via centrifugation at 4°C for 15 min, and the sample was then sterilized with 85% ethanol and dissolved in RNase-free water. The purity of the extracted RNA was confirmed by measuring absorbance at 230 nm and 260 nm using a spectrophotometer (ND-100, Nanodrop Technologies Inc., Wilmington, DE, USA), and only RNA samples with purity (optical density value of 230 nm/260 nm) measurements greater than 1.8 were selected and stored at −70°C until the experiment was carried out.

To synthesize cDNA, 1 μg of RNA and 1 μL each of oligo-dT (Invitrogen, USA) and RNase-free water were added. RNA was denatured at 80°C for 3 min, and cDNA was synthesized using 4 μL of 5×RT (reverse transcription) buffer, 5 μL of 2 mM dNTPs, 0.5 μL of RNase inhibitor (Promega, Madison, WI, USA), and 1 μL of moloney-murine leukemia virus RT (Promega, USA).

### RT-PCR and real time-qPCR

NCBI (http://www.ncbi.nlm.nih.gov) and the Ensembl Genome Browser (www.ensembl.org) were utilized to retrieve gene sequence information. The primers for amplification of myokines and myogenic marker mRNA (Table 1) were synthesized using PRIMER3 software (http://bioinfo.ut.ee/primer3-0.4.0/). Reverse transcriptase-PCR and real-time qPCR reactions were carried out in a 25 μL reaction solution using a C1000 Thermal Cycler (Bio Rad, Hercules, CA, USA) to measure the relevant expression of target genes. The solution was prepared as follows: 2 μL diluted cDNA (50 ng/μL) was added to 14 μL SYBR green master mix (Bio Rad, USA) and 1 μL each of 5 pmol/μL diluted forward and reverse primers. The conditions used for the real-time qPCR were as follows: initial denaturation at 94°C for 10 mins followed by 40 cycles of denaturation at 94°C for 10 s, annealing at 60°C for 10 s, and extension at 72°C for 30 s. All measurements were carried out in triplicate for each specimen, and the 2^−ΔΔCt^ method was used to determine relative gene expression. The relative expression of target genes was normalized with glyceraldehyde-3-phosphate dehydrogenase.

### MTT assay

Cell viability was assayed by measuring blue formazan that was metabolized from 3-(4,5-dimethylthiazol-2-yl)-2,5-diphenyltetrazolium bromide (MTT) by mitochondrial dehydrogenase. Horse muscle cells were re-suspended in the medium one day before H_2_O_2_ (Junsei, Japan) treatment, at a density of 2×10^5^ cells per well in 24-well culture plates. Liquid medium was replaced with fresh medium containing dimethyl sulfoxide (DMSO) for control. Horse muscle cells were incubated with various concentrations of H_2_O_2_. MTT (5 mg/mL) was added to each well and incubated for 4 h at 37°C. The formazan product formed was dissolved by adding 200 μL DMSO to each well, and the absorbance was measured at 570 nm on an Ultra Multifunctional Microplate Reader (TECAN, Durham, NC, USA). All measurements were performed in triplicate and repeated at least three times.

### Statistical analysis

Means and standard deviations of gene expression were calculated using Microsoft Excel. The statistical significance (* p<0.05, ** p<0.01, or *** p<0.001) was assessed using an analysis of variance, followed by an unpaired sample t-test using the Prism 5 program (San Diego, CA, USA).

## RESULTS

### Expression of exercise-related myokines before and after exercise in horses

**Figure 1:**
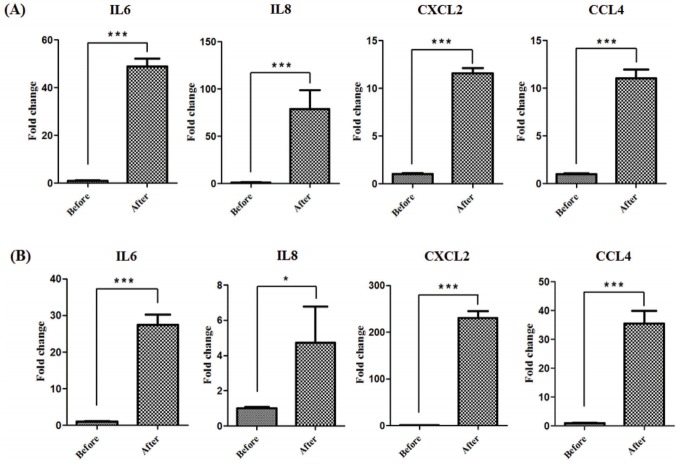
The effect of exercise on myokine expression in the horse skeletal muscle tissue. Expression of myokines (*IL-6*, *IL-8*, *CXCL2*, and *CCL4*) before and after exercise in Thoroughbred (A) and Jeju (B) horses was identified by quantitative PCR. The results were normalized to glyceraldehyde-3-phosphate dehydrogenase. Error bars represent standard deviation (n = 3), * p<0.05, *** p<0.001. *IL6*, interleukin 6; *IL8*, interleukin 8; *CXCL2*, (*C-X-C motif*) ligand 2; *CCL4*, (*C-C motif*) ligand 4; PCR, polymerase chain reaction.

To examine the expression patterns of horse myokines including *IL-6*, *IL-8*, *CXCL2*, and *CCL4* after exercise, mRNA levels were quantified in muscle tissues from Thoroughbred horses following one bout of trotting on the treadmill. Expression of myokines was significantly increased after exercise in Thoroughbreds (*** p<0.001, Figure 1A). Similar expression patterns were observed in the muscles of Korean native Jeju horses, demonstrating that regulation of myokine expression by exercise is conserved in horses (Figure 1B).

### Culture and validation of primary skeletal muscle cells of horse

**Figure 2:**
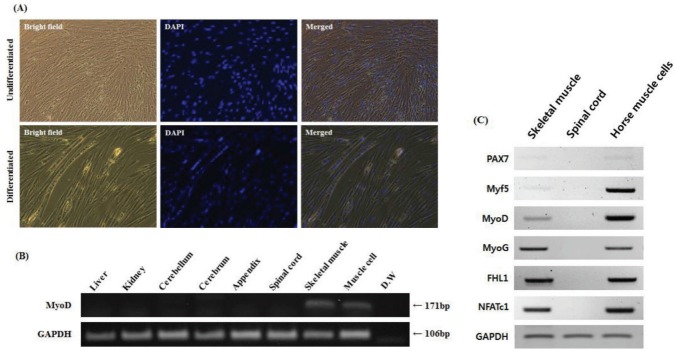
Characterization of horse skeletal muscle cells with myotube formation and myogenic marker expression. (A) Morphology of undifferentiated and differentiated horse skeletal muscle cells (magnification: 200×). Undifferentiated horse muscle cells show mononuclei. Multinuclear cells emerged after culture in differentiated media for 12 days. 4′,6-diamidino-2-phenylindole (DAPI) staining was used to determine the nuclei. (B) *MyoD* expression was identified by RT-PCR in horse tissues including liver, kidney, cerebellum, cerebrum, appendix, spinal cord, skeletal, and horse muscle cells. *GAPDH* was used as a reference gene. (C) Reverse transcriptase-polymerase chain reaction analysis of myogenic marker expression in the skeletal muscle tissue, spinal cord, and muscle cells of horses. *GAPDH* was used as a reference gene. *MyoD*, myoblast determination protein; *GAPDH*, glyceraldehyde-3-phosphate dehydrogenase; *PAX7*, paired box 7; *Myf5*, myogenic factor 5; *MyoG*, myogenin; *FHL1*, four and a half LIM domains 1; *NFATc1*, nuclear factor of activated T cells 1; D.W, distilled water.

We isolated and cultured muscle cells from the skeletal muscle tissue of a neonatal Thoroughbred to establish a reliable system that allowed an *in vitro* study in horses. Horse muscle cells were stably maintained even after passage 23 in Medium 199 supplemented with 10% FBS and 2% DES. It is interesting to note that horse muscle cells were larger than those of mouse muscle cell line C2C12. To test whether horse primary myoblast cells possess the capacity for differentiation into myotube cells, horse muscle cells with 80% confluence were cultured in Medium 199 supplemented with 2% FBS for 12 days. During myogenic differentiation, myoblasts were fused into multi-nucleated fibers (Figure 2A). Subsequently, we conducted RT-PCR for *MyoD* (one of the myogenic markers) expression to confirm the origin of the cells. As a result, muscle-specific transcription factor *MyoD* was specifically expressed in both skeletal muscle tissues and cultured cells, but it was not expressed in the other tissues (Figure 2B). RT-PCR analysis for myogenic markers including paired box 7 (*PAX7*), myogenic factor 5 (*Myf5*), myogenin (*MyoG*), four and a half LIM domains 1 (*FHL1*), and nuclear factor of activated T cells 1 (*NFATc1*) confirmed that horse muscle cells maintained myogenic features *in vitro* (Figure 2C).

### Myokine expression in oxidative stress-induced horse muscle cells

**Figure 3:**
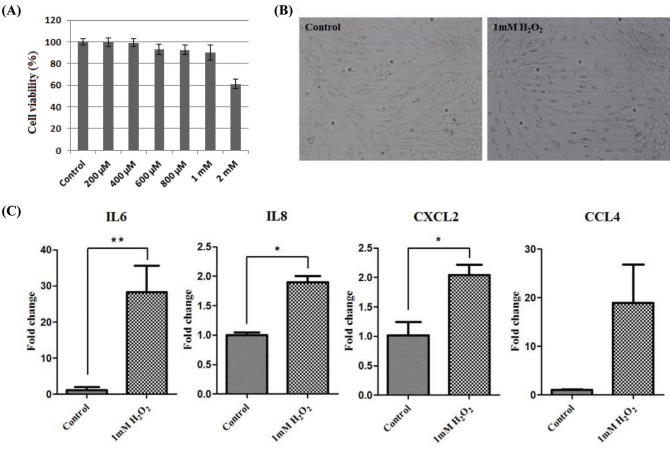
The effect of oxidative stress on myokine expression in the horse muscle cells. (A) MTT assay to measure cell viability in the horse muscle cells after treatment with H2O2 at different concentrations for 6 h. The x-axis indicates that the concentration of H_2_O_2_. (B) Phenotypic comparison between regular and 1 mM H_2_O_2_-treated horse skeletal muscle cells (magnification: 100×). (C) Expression of myokines (*IL-6*, *IL-8*, *CXCL2*, and *CCL4*) after 1 mM H_2_O_2_ treatment for 6 h was identified by quantitative polymerase chain reaction. The results were normalized to *GAPDH*. Error bars represent standard deviation (n = 3), * p<0.05, ** p<0.01. *IL-6*, interleukin 6; *IL-8*, interleukin 8; *CXCL2*, (*C-X-C motif*) ligand 2; *CCL4*, (*C-C motif*) ligand 4, *GAPDH*, glyceraldehyde-3-phosphate dehydrogenase.

To induce oxidative stress, horse muscle cells were treated with H_2_O_2_ for 6 h at different concentrations. The effect of oxidative stress with H_2_O_2_ at 200 μM to 2 mM on cell viability of horse muscle cells was assessed by the MTT assay. H_2_O_2_ at 2 mM reduced cell viability by approximately 60% compared to control (Figure 3A). To maximize oxidative stress, horse muscle cells were exposed to 1 mM H_2_O_2_ for 6 h. There were no morphological changes in H_2_O_2_-treated horse muscle cells (Figure 3B). Then, the effects of oxidative stress on the expression of the myokine genes was evaluated by qRT-PCR. Oxidative stress caused by H_2_O_2_ treatment increased the expression of *IL-6* (** p<0.01), *IL-8* (* p<0.05), and *CXCL2* (* p<0.05) mRNA, but *CCL4* expression was not altered significantly (Figure 3C).

## DISCUSSION

A variety of cytokines are secreted from muscle cells after exercise [19,24], and this creates a milieu for muscle recovery and inflammatory responses. Although the horse is a representative model for exercise, the expression of exercise-induced cytokines has been poorly studied in horses. Several studies have investigated cytokine expression following exercise [24], and the effects of exercise-induced cytokines in muscles have been observed in previous studies, which found that exercise greatly reduces the risk of chronic inflammatory diseases [25]. Physical activity induces reactive oxygen species (ROS), which initiate signaling cascades and exert various functions [26]. In addition to the expression of oxidative stress-related genes, ROS are closely related to inflammation induction [26]. Inflammatory cytokines promote the involvement of immune cells such as macrophages or neutrophils [27]. It is assumed that exercise-induced inflammatory responses are required for regenerative and adaptive processes in the skeletal muscle. In this study, we examined the expression of myokines after horse exercise, and *IL-6*, *IL-8*, *CXCL2*, and *CCL4* increased after exercise in Thoroughbred and Jeju horses (Figure 1). It has been previously shown that the exercise-induced *IL-6* has specific roles in fat metabolism and immune system [16,17]. Although the exact function of *IL-8*, *CXCL2*, and *CCL4* in exercise still remains uncertain, the increased expressions after exercise in muscle tissue are similar among species [11,12,19]. Therefore, these results indicate that exercise-related myokines may play a role in exercise regardless of species and breed.

In this study, we established a culture system for horse muscle cells derived from the skeletal muscle tissue of a neonatal Thoroughbred (Figure 2A). After 23 culture passages *in vitro*, we investigated whether these cells possessed the myogenic features by examining myogenic markers using RT-PCR. *PAX7* is regarded as an important gene for the specification of myogenic satellite cells [28]. Though *PAX7* was expressed weakly in both horse skeletal muscle tissue and horse muscle cells, it was reasonable to assume that *PAX7* may play a role in the specification of myogenic cell lineages in horses. In addition, other myoblast markers, *Myf5*, *MyoD*, *MyoG*, *FHL1*, and *NFATc1*, were expressed in horse muscle cells, indicating that the cell population possesses myogenic features observed in other mammals; however, further investigation is required (Figure 2C). Because the cells were derived from neonatal skeletal muscle tissue, they may contain various myogenic cells. Further study is needed to demonstrate the exact state of these horse skeletal muscle-derived cells. Finally, we validated the possibility of a suitable *in vitro* system for studying horse muscle physiology and exercise-induced muscle disease using *in vitro* cultured horse muscle cells (Figure 3). Horse muscle cells developed in this study will provide an important system for the functional study of exercise-related genes.

In conclusion, exercise can induce the expression of cytokines, which play an important role in muscle regeneration and anti-inflammation in horse skeletal muscle tissues after exercise. Additionally, we established a horse skeletal muscle cell culture, which will be a valuable tool for investigating the expression and function of exercise-related genes.

## Figures and Tables

**Table 1:** List of primers for myokine and myogenic marker detection

Gene	Accession number	Sequence (5′ to 3′)	Annealing Tm (°C)	Product size (bp)
*IL-6*	NM_001082496.2	Forward: CACCACTGGTCTTTCGGAGT	60	156
		Reverse: TCAGGGGTGGTTACTTCTGG		
*IL-8*	NM_001083951.2	Forward: GCTTTCTGCAGCTCTGTGTG	58	153
		Reverse: TCTGAGTTTTCGCAGTGTGG		
*CXCL2*	NM_001143955.1	Forward: CAAGAACATCCAGAGCGTGA	60	154
		Reverse: GCTGCCCTTCTTTAGCATCTT		
*CCL4*	XM_001503888.4	Forward: CTCTCTCTCCTCGTGCTGGT	60	151
		Reverse: AGAGGCTGCTGGTCTCGTAA		
*MyoD*	NM_001317253.1	Forward: GACGGCATGATGGACTACAG	63	171
		Reverse: GGGACTCTCGGTGGAGATG		
*PAX7*	NM_001317254.1	Forward: CTATCAGGAGACGGGGTCCA	60	434
		Reverse: GTGTCACAGCATTAGCCTCCT		
*Myf5*	NM_001302119.1	Forward: TTTCGGGGACGAGTTTGAGC	60	445
		Reverse: CCAGACGGGGCTGTTACATT		
*MyoG*	NM_014855253.1	Forward: GCGTGCAAGGTGTGTAAGAG	60	415
		Reverse: TGTCCACAATGGAGGTGAGC		
*FHL1*	XM_023634306.1	Forward: TAAGAACCGCTACTGGCACG	60	497
		Reverse: GAGGAGCTGCTAAGCTTCGAT		
*NFATc1*	XM_02364022.1	Forward: TTGGTGGTTGAGATACCGCC	60	351
		Reverse: AGAAAGGTCGTGGAGCTTCG		
*GAPDH*	NM_001163856.1	Forward: GGTGAAGGTCGGAGTAAACG	60	106
		Reverse: AATGAAGGGGTCATTGATGG		

*IL-6*, interleukin-6; *IL-8*, interleukin-8; *CXCL2*, chemokine (*C-X-C motif*) ligand 2; *CCL4*, chemokine (C-C motif) ligand 4; *MyoD*, myoblast determination protein; *PAX7*, paired box 7; *Myf5*, yogenic factor 5; *MyoG*, myogenin; *FHL1*, four and a half LIM domains 1; *NFATc1*, nuclear factor of activated T cells 1; *GAPDH*, glyceraldehyde-3-phosphate dehydrogenase.
